# Prophylaxis

**Published:** 1907-05

**Authors:** 


### PROPHYLAXIS.

(Abstract of Paper Read Before Iowa State Society May 7, 1907.)

Dr. D. D. Smith, of Philadelphia, Pa., among other instructive papers, gave an interesting and practical paper on "Oral Prophylaxis." The treatment, to begin with, was discovered, like so many other things of benefit to mankind, by accident.

A nervous child two and a half years old whose teeth were showing every sign of speedy decay was treated once a week by the lecturer with orange stick and pumice stone. This was followed up until every sign of decay had disappeared and the child at the proper time shed her teeth with no irregularities whatever of nature. Subsequently a perfect mouthful of permanent teeth was the result.

Dentistry is not respected as it should be by the medical profession because their motto is not to "help humanity and stop decay" as it should be. If dentists would adopt this prophylaxis treatment on patients, keeping the teeth smooth by polishing once in four weeks, not only teeth would be benefited, but a number of troubles under the jurisdiction of the medical profession would be avoided. Tonsilitis, for instance, can and has been not only avoided but cured by the prophylaxis treatment. Pyorrœa also by the removal of infections along the gums, this treatment making them tense and hard. Sore throat will be less frequent also. Take two hundred children—relative conditions and surroundings being generally the same—one hundred subjected to Dr. Smith's treatment will, all of them, it is safe to say, reach maturity safely. The other hundred not so treated, seven of them will have tuberculosis.

It is stated by Dr. Smith that 75 per cent if not 90 per cent decay of teeth will be avoided if prophylaxis is used.

Statistics show that out of 11,000 recruits for the Boer war only 3,000 were eccepted. The other 8,000 being rejected presumably on account of the condition of the teeth.

Surely, indeed, with such a record of an enlightened people, is it not time that work should be done in the line of prophylaxis as advocated by Dr. Smith?

A discussion followed, led by Dr. Warren, of Missouri Valley. Dr. Warren thought Dr. Smith's treatment very efficacious, but too metropoltian for the ordinary practitioner. Dr. Warren also advocated rinsing of mouth, after meals, and also emphasized the harm of "nibbling" between meals.—Am. Dental Jour.

